# The effect of sirolimus on angiomyolipoma is determined by decrease of fat-poor compartments and includes striking reduction of vascular structures

**DOI:** 10.1038/s41598-021-87930-4

**Published:** 2021-04-19

**Authors:** Elieser Hitoshi Watanabe, Fernando Morbeck Almeida Coelho, Hilton Leão Filho, Bruno Eduardo Pedroso Balbo, Precil Diego Miranda de Menezes Neves, Fernanda Maria Franzin, Fernando Ide Yamauchi, Luiz Fernando Onuchic

**Affiliations:** 1 Division of Nephrology, Department of Medicine, University of São Paulo School of Medicine, Avenida Dr. Arnaldo, 455 - Sala 4304, São Paulo, 01246-903 Brazil; 2 Division of Radiology, Department of Radiology and Oncology, University of São Paulo School of Medicine, São Paulo, Brazil

**Keywords:** Urological cancer, Medical genetics, Kidney diseases, Urological manifestations

## Abstract

Renal angiomyolipomas hemorrhage is associated with their size and vascular constitution. The effects of sirolimus on different components of angiomyolipomas was analyzed in patients with tuberous sclerosis complex, sporadic lymphangioleiomyomatosis and multiple sporadic angiomyolipomas. Thirty angiomyolipomas from 14 patients treated with sirolimus were retrospectively evaluated. A Hounsfield-unit threshold was used to classify angiomyolipomas in fat-rich, fat-poor and intermediate-fat tumors, and to categorize tumor compartments in fat rich, fat poor, intermediate fat and highly vascularized. Diameter variations were measured to assess the effects on aneurysmatic/ectatic vascular formations. Volume reduction following treatment with sirolimus was higher in fat-poor than fat-rich angiomyolipomas. Tumor reduction was mainly determined by decrease of the fat-poor and highly-vascularized compartments while the volume of the fat-rich compartment increased. Broad liposubstitution was observed in some tumors. A median reduction of 100% (75 to 100) in the diameter of aneurysmatic/ectatic vascular structures was observed. Our study showed that sirolimus reduces the size of angiomyolipomas by decreasing primarily their highly-vascularized and fat-poor compartments. This effect is associated with a remarkable reduction of tumoral aneurysms/ectatic vessels, revealing the likely mechanism responsible for the risk-decreasing effect of mTOR inhibitors on angiomyolipoma bleeding. These findings support the role of mTOR in the development of angiomyolipoma blood vessels.

## Introduction

Renal angiomyolipomas (AMLs) are perivascular epithelioid tumors containing adipocyte-like, muscle-like and epithelioid cells as well as dysmorphic blood vessels^1,2^. AMLs affect up to 2.2% of general adult population^3^ and are usually sporadic, however approximately 10% of the cases are associated with tuberous sclerosis complex (TSC)^4^. TSC is characterized by the development of neoplasm in various organs and tissues, particularly skin, central nervous system, kidneys, lungs and heart^5,6^. Notably, AMLs have been reported in 49 to 60% of TSC patients evaluated by renal imaging^7,8^. AMLs are associated with lymphangioleiomyomatosis (LAM), which can manifest as sporadic (s-LAM) or TSC-associated (TSC-LAM) variants^9,10^. LAM affects ~ 30% of the TSC patients while 47 to 60% of s-LAM patients develop AMLs^11,12^.

Although small AMLs rarely cause relevant complications or symptoms, larger tumors may compress adjacent structures and lead to abdominal discomfort or pain^4,13,14^. AML-related complications also include kidney impingement, vena cava and retroperitoneal infiltration, and hemorrhage secondary to aneurysm rupture, a potentially lethal event^4,14–18^.

Sporadic and TSC-associated AMLs share a common molecular pathogenetic mechanism, characterized by loss of suppression of mammalian (mechanistic) target of rapamycin (mTOR)^19,20^. This signaling pathway promotes protein synthesis, cell hypertrophy and proliferation^19,21^. Its inappropriate hyperactivation, therefore, favors tumor development and growth. Based on this mechanism, clinical trials with mTOR inhibitors (mTORi) were carried out, showing striking reduction of AML size with acceptable safety profile^22–26^.

The effects of mTORi on the different AML components, however, are not well characterized. A recent study reported a heterogeneous volume-reducing effect of the mTORi everolimus on AMLs. In that study a more efficient reduction was observed in fat-poor tumors^27^, however, the effects on the different tumor compartments have not been assessed. Similarly, a previous study using magnetic resonance imaging (MRI) revealed increase in AML fat composition following treatment with everolimus, however the responses of specific AML components were not directly quantified^28^. Since hemorrhage is strongly associated with tumor vascularization and presence of intra-tumoral aneurysms larger than 0.5 cm^29,30^, the response of this compartment to mTORi is critical to understand the role of mTOR in AML vascular structure. Moreover, given that bleeding is the most relevant clinical aspect associated with AMLs, it is essential to evaluate the effect of these drugs on the patient’s bleeding risk. In this study, we present a description of retrospectively gathered computed tomography findings, which demonstrate the differential effects of mTORi on various tumor compartments.

## Methodology

### Study population and radiologic analyses

We retrospectively analyzed patients with multiple/large AMLs followed at the University of São Paulo School of Medicine Medical Center between April/2010 and June/2020, who had been treated with sirolimus for this condition. Among them, we selected subjects with pre- and post-contrast abdominal computed tomography (CTs) at baseline and after at least three months of treatment with sirolimus. All candidate patients for enrollment had the CTs performed in our medical center and had all the image sequences available in the hospital storage databank. All patients underwent abdominal computed tomography using 64-channel multidetector scanners. A routine protocol was performed with the patients in supine position. The slice thickness varied between 1 and 1.5 mm, with all images reconstructed in 2 to 2.5 mm. Intravenous contrast media was used in all cases using a routine dose of 1.3 mL/kg (maximum volume of 150 mL). The CT studies were reviewed on a dedicated workstation (ADW—Advanced Workstation—GE Healthcare). The clinical and laboratorial data were reviewed using the medical charts. Two certified abdominal radiologists reviewed the CT studies independently, blinded to clinical data and original radiology reports, except for the knowledge that each patient had renal AMLs treated with sirolimus. Interobserver variability was quantified based on data collection of five patients, including 13 AMLs, also performed by a second independent radiologist. This second analysis followed the entire established protocol, comprising the pre- and post-sirolimus treatment CTs. Notably, the interclass correlation index was very high: 0.998 for absolute concordance.

Since there is no established consensus on radiological classification of renal AMLs^31–33^, we created a novel classification based on previously published tissue-specific ranges of threshold attenuation. According to these studies, attenuation below − 30 HU is referred as fat^34–36^, while skeletal and smooth muscle have attenuation values ranging from 30 and 50 HU^36,37^. After contrast enhancement, the attenuation values of large blood vessels usually exceed 200 HU^38^; small vessels, however, show lower attenuation levels, displaying a threshold of 100 HU in previous studies^39,40^. These data imply that AML attenuation pixels < − 30 HU reflect fat whereas pixels with attenuations > 30 HU are consistent with absence of fat. In addition, since attenuation values corresponding to muscle do not reach 100 HU following contrast enhancement, and considering the AML components, pixels with attenuation > 100 HU represent primarily blood vessels and highly vascularized tumor portions.

In non-contrasted sequences we evaluated the AMLs based on the mean attenuation value corresponding to a region of interest (ROI) placed into the lesion, encompassing at least two thirds of its largest area in the axial acquisition. We classified the tumors in three categories: fat-rich (< − 30 HU; FRT), intermediate-fat (≥ − 30 and ≤ 30 HU; IFT) and fat-poor (> 30 HU; FPT). All patients were screened for FRT, IFT and FPT, having one representative lesion of each profile been selected for analysis whenever identified. Total volume response to sirolimus was quantified for all included tumors. The tumor total volume was calculated by free-hand delimitation of tumor contour for each CT slice, followed by 3D reconstruction. This tumor segmentation generated an attenuation histogram including all pixels contained in the tumor volume. The volume of each tumor compartment was calculated by counting the pixels within the respective attenuation interval (Fig. S1).

This evaluation was followed by corticomedullary-phase analysis, which allowed the measurement of such a response in specific AML compartments. Pixel densities below − 30 HU corresponded to fat-rich compartments (FRC), densities ≥ − 30 and < 30 HU were associated with intermediate-fat compartments (IFC), attenuation ≥ 30 and < 100 HU indicated a fat-poor compartment (FPC), and pixel densities ≥ 100 HU identified highly-vascularized compartments (HVC). This approach allowed the quantification of volume response to sirolimus for each of the AML compartments, characterizing its potentially differential effects upon each of the tumor components. To improve the assessment of sirolimus actions on the AML vascular structures, each of the analyzed tumors had the diameter of its largest aneurysmatic/ectatic vessel determined following the CT corticomedullary phase.

### Statistical analyses

Data on continuous variables were tested for normality using the Shapiro–Wilk test. Since this analysis revealed non-parametric distributions, these data are presented as median and 25 and 75 percentiles. Categorical data are expressed as absolute values and percentages. Non-parametric data were compared using the Mann–Whitney U test for two independent samples or the Wilcoxon signed rank test for two-time measures. Non-parametric data associated with multiple groups were compared using the Kruskal–Wallis test, while multiple comparisons were corrected applying the Bonferroni method.

Given the limited number of cases, multivariable analyses for binary endpoints were performed using logistic regression with Firth’s penalization^41^. Statistical significance was considered for asymptotic *P* < 0.05. The analyses were performed using SPSS 24.0, GraphPad Prism 8.0 and Stata 16.0.

### Ethical aspects

The current research project was carried out in accordance with relevant guidelines and regulations and had been approved by Ethics Committee for Analysis of Research Projects (CAPPesq) and National Research Ethics Committee (CONEP). Informed consent was not obtained by subjects. Since it was a retrospective study and patients were not exposed to any potential risk, it was given waiver for the need of informed consent by CONEP.

### Ethics approval

This work was approved by the HC-FMUSP Research Ethics Committee under the CAAE number 44709915.1.0000.0068.

## Results

### Baseline patient and angiomyolipoma features

We selected 14 patients that fulfilled all inclusion criteria: 11 with TSC, one with LAM and two with multiple sporadic AMLs. The patients’ median estimated glomerular filtration rate (eGFR) was 110.5 (69.3 to 120.8) mL/min/1.73 m^2^. All patients had an eGFR above 30 mL/min/1.73 m^2^ and had no report of complications related to iodine administration. Thirty AMLs from these patients were selected for analyses, since eight patients did not present all the AML fat profiles. The mean AML pre-treatment size was 4.3 cm (2.8 to 7.0). The baseline features of analyzed patients and respective tumors are depicted in Table 1.

**Table 1:** Characterization of patients and angiomyolipomas.

	n	Median (25 to 75%) or n (%)
**Patients**		
Age	14	28.4 (20.4 to 47.3)
Sex		
M/F	14	4 (28.8)/10 (71.4)
TSC		11 (78.6)
TSC-LAM	11	4 (36.4)
Sporadic AML		3 (21.4)
Sporadic LAM		1 (7.1)
Sporadic multiple AMLs		2 (14.3)
Serum creatinine (mg/dL)	14	0.76 (0.70 to 1.10)
eGFR (mL/min/1.73 m^2^)	14	110.5 (69.3 to 120.8)
Sirolimus serum level (ng/mL)	10	7.9 (6.1 to 10.1)
Time of treatment before CT (days)	14	304.5 (203.5 to 418.0)
**AMLs**		
Pre-treatment tumor size (mm)	30	42.9 (28.0 to 70.3)
Pre-treatment aneurysm diameter (mm)	20	3.0 (1.0 to 5.0)

*AML* angiomyolipoma, *CT* computed tomography, *eGFR* estimated glomerular filtration rate, *LAM* lymphangioleiomyomatosis, *M/F* male/female, *TSC* tuberous sclerosis complex.

### TSC- and sporadic AMLs show similar reduction in response to sirolimus

Twenty-five TSC- and five sporadic AMLs presented pre-treatment diameters of 2.9 cm (2.0 to 5.7) and 4.8 cm (3.5 to 7.1), respectively, and pre-treatment volumes of 11.3 cm^3^ (3.2 to 76.1) and 33.5 cm^3^ (11.5 to 107.6), respectively. Both diameter and volume did not significantly differ between TSC- and sporadic AMLs. The median percent response in volume following treatment with sirolimus was − 49.0% (− 65.5 to − 9.5) and − 68.5% (− 75.4 to − 27.3) for TSC- and sporadic-AMLs, respectively, also revealing no significant difference between the two groups (Fig. S2). Since the baseline features and responses to treatment were not significantly different between the two groups, we added the five sporadic AMLs to the 25 TSC-AMLs to compose the total AML sample whose response to sirolimus was analyzed.

### Sirolimus reduces tumor volume more effectively in fat-poor AMLs

**Figure 1:**
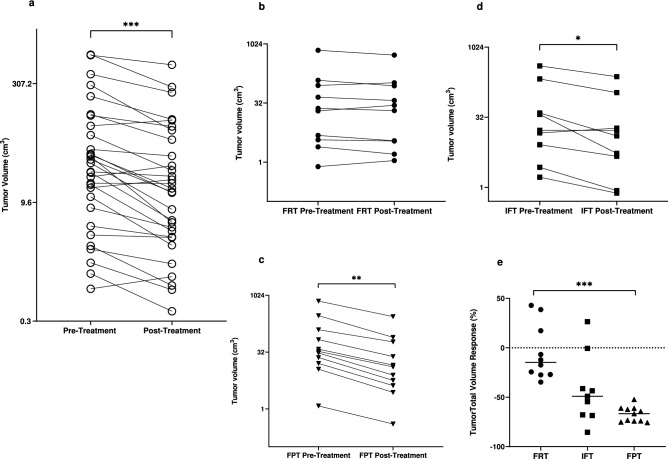
Total tumor volume pre- and post-treatment with sirolimus for all AMLs (**a**), fat-rich tumors (**b**), intermediate-fat tumors (**c**) and percent response to sirolimus for each tumor type (**d**). Repeated measures were analyzed using the Wilcoxon test, whereas comparisons among tumor fat profiles were performed with the Kruskal–Wallis test. *AML* angiomyolipoma, *FPT* fat-poor tumor, *FRT* fat-rich tumor, *IFT* intermediate-fat tumor. *P < 0.05, ***P < 0.001.

**Figure 2:**
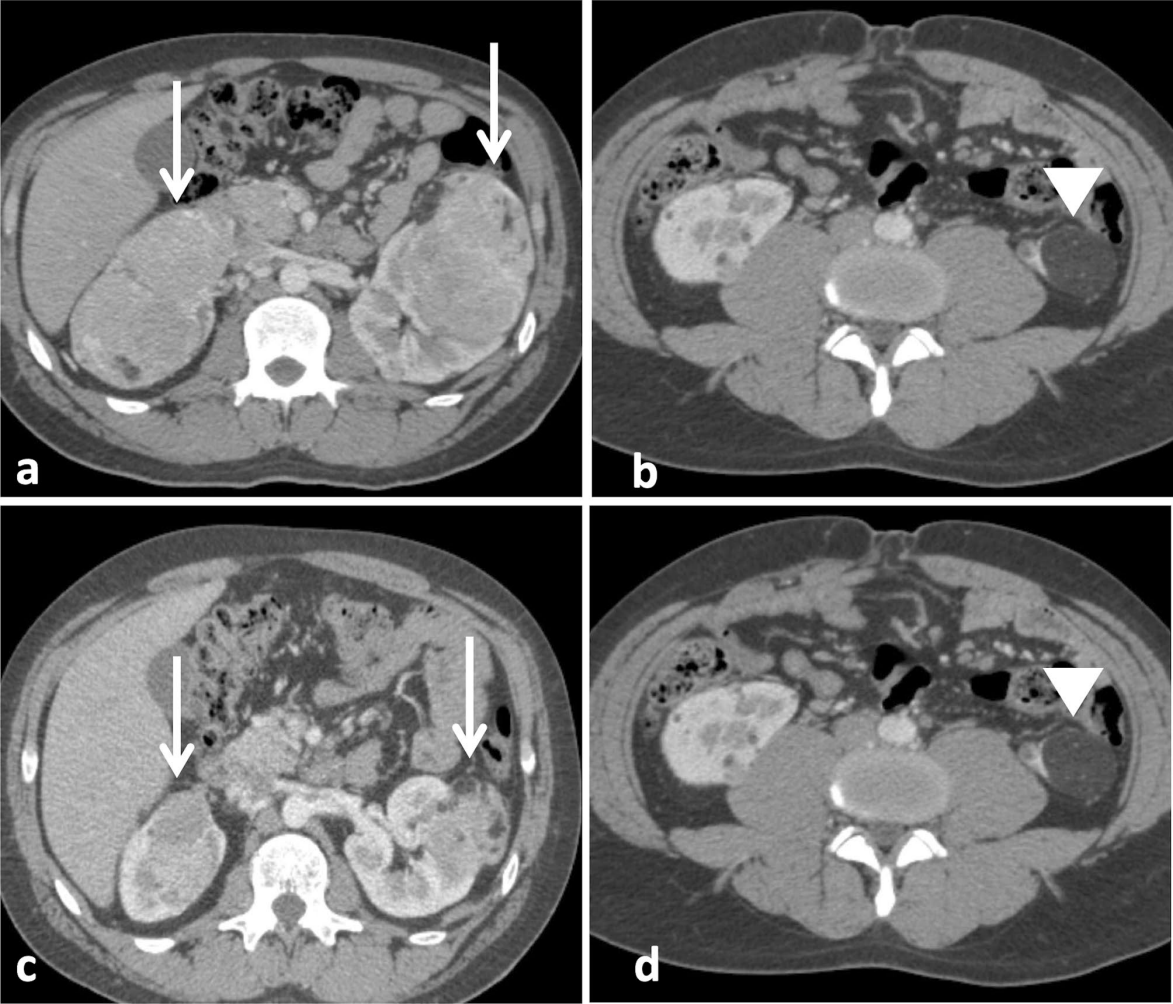
Response of renal AMLs to treatment with sirolimus. (**a**,**b**) Axial corticomedullary-phase CT images obtained before treatment show lobulated fat-poor AMLs in both kidneys (arrows) and a fat-rich AML arising from the lower pole of the left kidney (arrowhead). (**c**,**d**) Axial corticomedullary-phase CT images acquired after 7 months of treatment show significant volumetric reduction of the fat-poor AMLs (arrows) and nonsignificant change in the fat-rich AML (arrowhead).

As expected, sirolimus effectively reduced tumor volume, leading to a median AML volume reduction of − 50.6% (− 68.0 to − 16.1) (Fig. 1a). The tumoral volume response, however, was heterogeneous among the different tumor fat profiles. FRTs presented milder responses to sirolimus than FPT [− 14.8% (− 27.3 to 22.6) versus − 66.7% (− 74.3 to − 61.2), *P* < 0.001] (Fig. 1b,c,e and 2). IFTs, in turn, displayed a volume decrease of − 49.0% (− 68.2 to 21.0), a value not significantly different from the FRT and FPT groups (Fig. 1d and Table 2).

**Table 2:** Pre- and post-treatment volumes of angiomyolipomas and percent responses according to the tumor fat profile.

	Median (25 to 75%)				
	FRT n = 10	IFT n = 9	FPT n = 11	*P*	All n = 30
Pre-treatment diameter (cm)	39.0 (22.0 to 70.8)	48.0 (24.5 to 73.0)	45.7 (38.0 to 74.0)	0.815	42.9 (28.0 to 70.3)
Pre-treatment volume (cm^3^)	21.8 (3.4 to 97.6)	16.8 (5.5 to 126.0)	33.5 (16.1 to 125.4)	0.651	26.9 (7.4 to 97.6)
Post-treatment volume (cm^3^)	24.3 (3.0 to 92.3)	12.7 (2.7 to 63.5)	13.0 (4.2 to 59.9)	0.851	13.7 (3.5 to 65.6)
Percent response (%)	− 14.8 (− 27.2 to 22.6)^a^	-49.0 (− 68.2 to − 21.0)	− 66.7 (− 74.2 to − 61.2) ^a^	< 0.001	− 50.6 (− 68.0 to − 16.1)

Comparisons among FRT, IFT and FPT were performed with Krusk l-Wallis test. Post-test significance was corrected using the Bonferroni method. ^a^P < 0.001 in the post-test analysis.

*FPT* fat-poor tumor, *FRT* fat-rich tumor, *IFT* intermediate-fat tumor.

### AML fat poorness predicts a better response to sirolimus

Using the Firth’s logistic regression for an at-least-50% response of volume reduction, we evaluated fat profile, gender, age, serum sirolimus level, treatment length and tumor size at baseline as potential predictors of volume response to sirolimus. This analysis identified the FPT pattern as the sole predictor of an at-least-50% decrease in tumor volume following this treatment (Table 3).

**Table 3:** Firth’s logistic regression for an at-least-50% tumor volume reduction.

Variable	Odds ratio	P	95% CI
AML fat profile			
FRT	1		
IFT	10.5	0.164	0.4 to 289.9
FPT	470.9	0.021	2.5 to 89,038.0
Sex: female	3.1	0.554	0.1 to 135.7
Age	1.1	0.206	1.0 to 1.2
Sirolimus serum level	0.7	0.307	0.4 to 1.3
Treatment length	1.0	0.242	0.9 to 1.0
Tumor size at baseline	1.0	0.802	0.9 to 1.0

*CI* confidence interval, *FPT* fat-poor tumor, *FRT* fat-rich tumor, *IFT* intermediated-fat tumor.

### Sirolimus induces significant reduction of AML vascular structures and is essentially not effective in the fat-rich compartment

**Figure 3:**
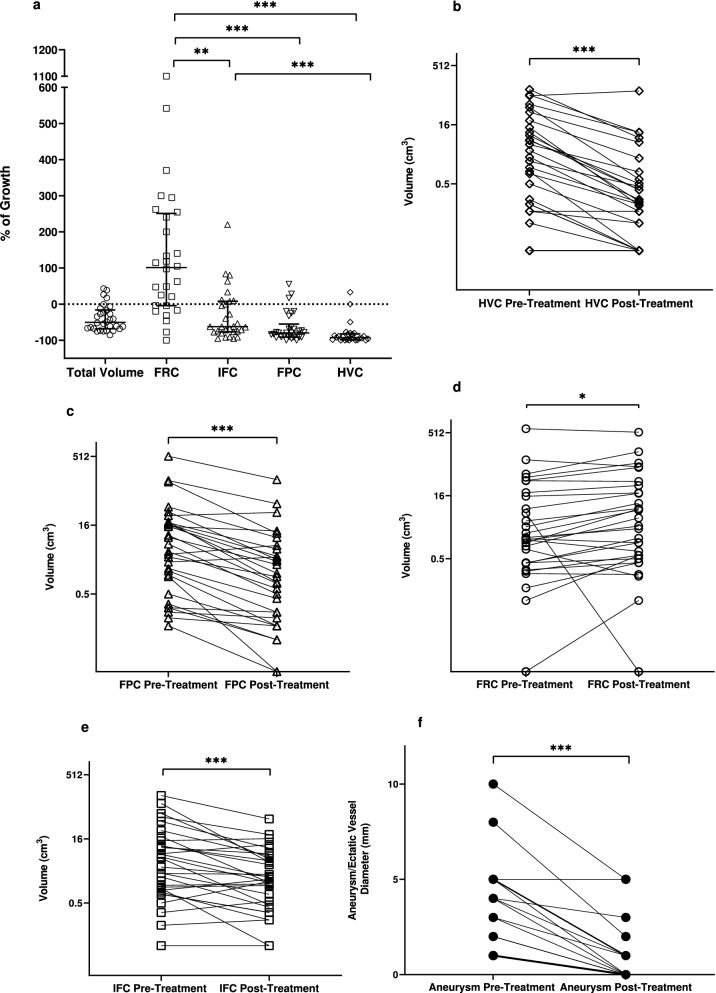
Percent variation of total tumor volume and volumes of tumor compartments following treatment with sirolimus (**a**). Variation of tumor compartment volumes—HVC (**b**), FPC (**c**), FRC (**d**) and IFC (**e**)—following treatment with sirolimus; and diameter variation of intra-tumoral ectatic vessels/aneurysms (**f**) in response to sirolimus. HVC, FPC and IFC presented significant volume reduction while FRC displayed volume increase. Comparisons between different groups were performed with Kruskal–Wallis test and repeated measures were analyzed with the Wilcoxon test. *FPC* fat-poor compartment, *FRC* fat-rich compartment, *HVC* highly-vascularized compartment, *IFC* intermediate-fat compartment. *P < 0.05, **P < 0.01, ***P < 0.001.

**Figure 4:**
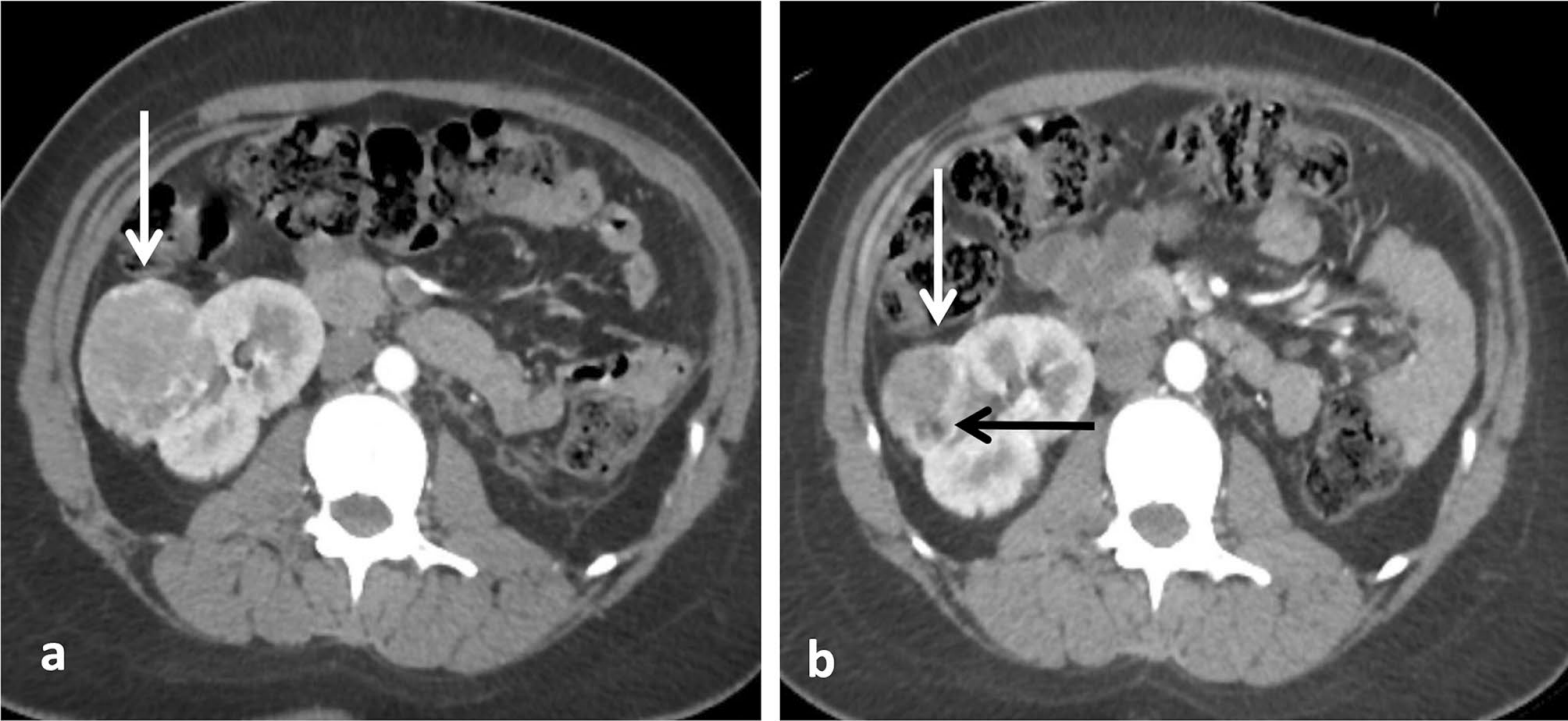
Change in AML volume and fat-containing pattern in response to sirolimus. (**a**) Pre-treatment axial corticomedullary-phase CT scan shows a right renal fat-poor AML (white arrow). (**b**) Post-treatment axial corticomedullary-phase CT image reveals tumor volume reduction (white arrow) while the fat component becomes visible (black arrow). Note that the AML total volume reduction (from 76.4 cm^3^ to 26.9 cm^3^) occurred due to diminishment of the fat-poor (from 60.1 cm^3^ to 13.0 cm^3^) and highly-vascularized (from 15.4 cm^3^ to 11.3 cm^3^) compartments.

**Figure 5:**
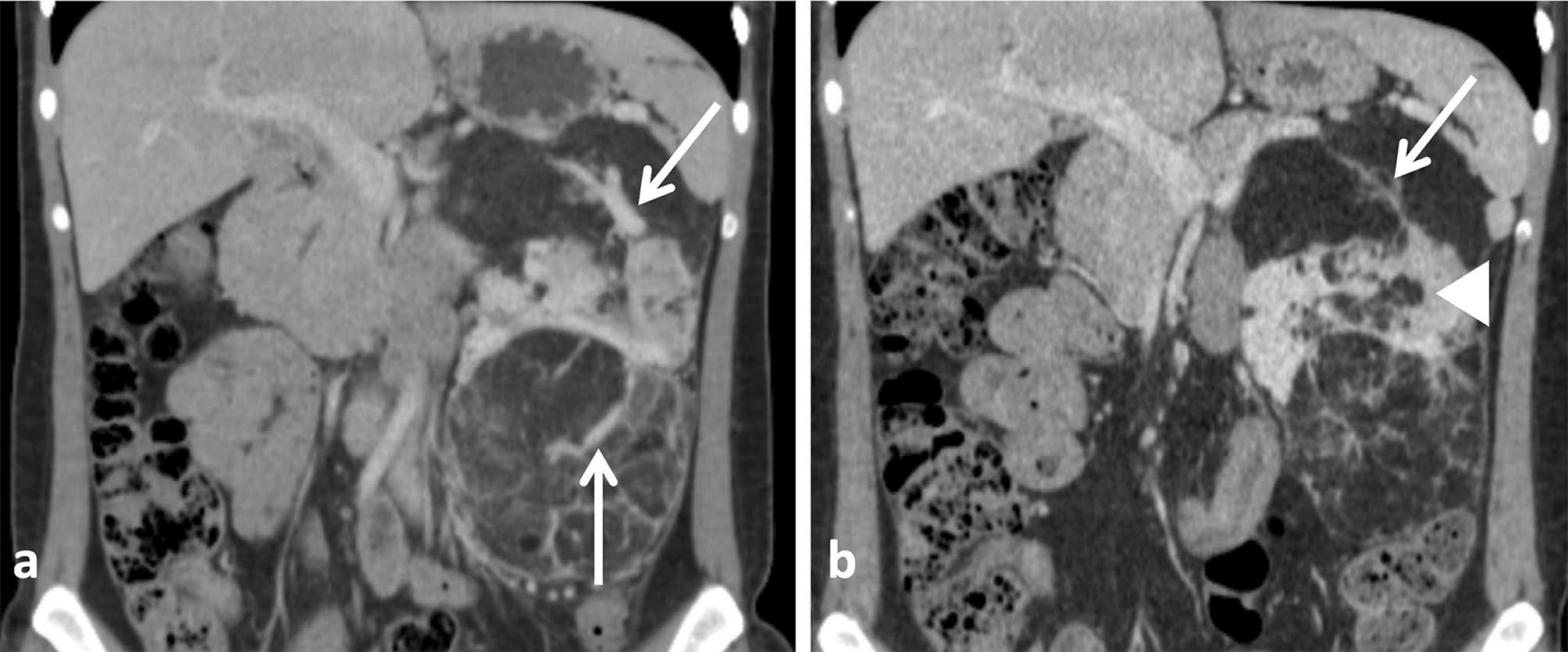
Response of intralesional vascular structures to sirolimus. Coronal nephrographic-phase CT images obtained (**a**) before the treatment and (**b**) after the treatment show disappearance of a left kidney large intralesional vascular structure and remarkable reduction of an intralesional aneurysm in response to treatment with sirolimus (arrows). An increase in the fat component is also seen (arrowhead).

Volume analyses of the different AML compartments following sirolimus revealed that all components, except for FRC, responded to treatment with significant volume reduction (Figs. 3a and 4, Table 4). Notably, HVC presented a remarkable volume decrease following this therapy [2.1 cm^3^ (0.1 to 15.2) vs. 0.16 cm^3^ (0.0 to 0.7), *P* < 0.001] (Fig. 3b), an effect that represented a 93.0% (82.3 to 98.6) volume reduction among the evaluated AMLs (Fig. 3a). The variations in ectatic vessel/aneurysm size were consistent with this finding, revealing disappearance or significant reduction in most of the assessed vascular lesions [3.0 mm (1.0 to 5.0) vs. 0.0 mm (0.0 to 1.0), *P* < 0.001] (Figs. 3f and 5), reflected in a median decrease in diameter of 100% (75 to 100).

**Table 4:** Pre- and post-treatment volumes of angiomyolipoma compartments and percent responses to treatment with sirolimus.

	Median (25 to 75%)			
	Response (%)	Pre-treatment volume (cm^3^)	Post-treatment volume (cm^3^)	*P*
FRC	101.2 (− 4.4 to 101.2)	1.5 (0.4 to 16.4)	2.8 (0.5 to 21.0)	0.023
IFC	− 62.5 (− 76.8 to − 62.5)	4.6 (1.0 to 15.4)	1.7 (0.4 to 5.8)	< 0.001
FPC	− 79.9 (− 91.3 to − 79.9)	5.2 (1.0 to 18.3)	1.1 (0.1 to 5.0)	< 0.001
HVC	− 93.0 (− 98.6 to − 93.0)	2.1 (0.1 to 15.2)	0.2 (0.0 to 0.7)	< 0.001

Comparisons between pre- and post-treatment volumes were performed with the Wilcoxon signed rank test.

*FPC* fat-poor compartment, *FRC* fat-rich compartment, *HVC* highly-vascularized compartment, *IFC* intermediate-fat compartment.

As expected based on the robust response of fat-poor AMLs to sirolimus, FPC presented significant volume reduction following this treatment [5.2 cm^3^ (1.0 to 18.3) to 1.1 cm^3^ (0.1 to 5.0), *P* < 0.001] (Fig. 3c), a volume decrease response of − 79.9% (− 91.3 to − 55.3) among the tumors (Fig. 3a). The AML compartment with attenuation of 30 HU or more was, in fact, responsible for 95.1% (46.4 to 100.0) of total tumor reduction.

Surprisingly, FRC volume not only did not decrease in response to sirolimus but also substantially increased in most cases. FRC volume, in fact, grew from 1.5 cm^3^ (0.4 to 16.4) to 2.8 cm^3^ (0.5 to 21.0) following this treatment (*P* = 0.023; Fig. 3d), an effect reflected in a 101.2% growth (− 4.4 to 250.9) (Fig. 3a).

Consistently with the aforementioned findings, IFC volume responded to treatment with sirolimus with an intermediate behavior between FRC and FPC. Indeed, the IFC volume decreased from 4.6 cm^3^ (1.0 to 15.4) to 1.7 cm^3^ (0.4 to 5.8) with treatment (*P* < 0.001, Fig. 3e), a response also expressed as a − 62.5% (− 76.8 to 8.1) volume reduction among the analyzed AMLs (Fig. 3a). It must be noted, however, that the IFC volume change was significantly different from the FRC one but did not significantly differ from the FPC volume variation (Fig. 3a).

**Figure 6:**
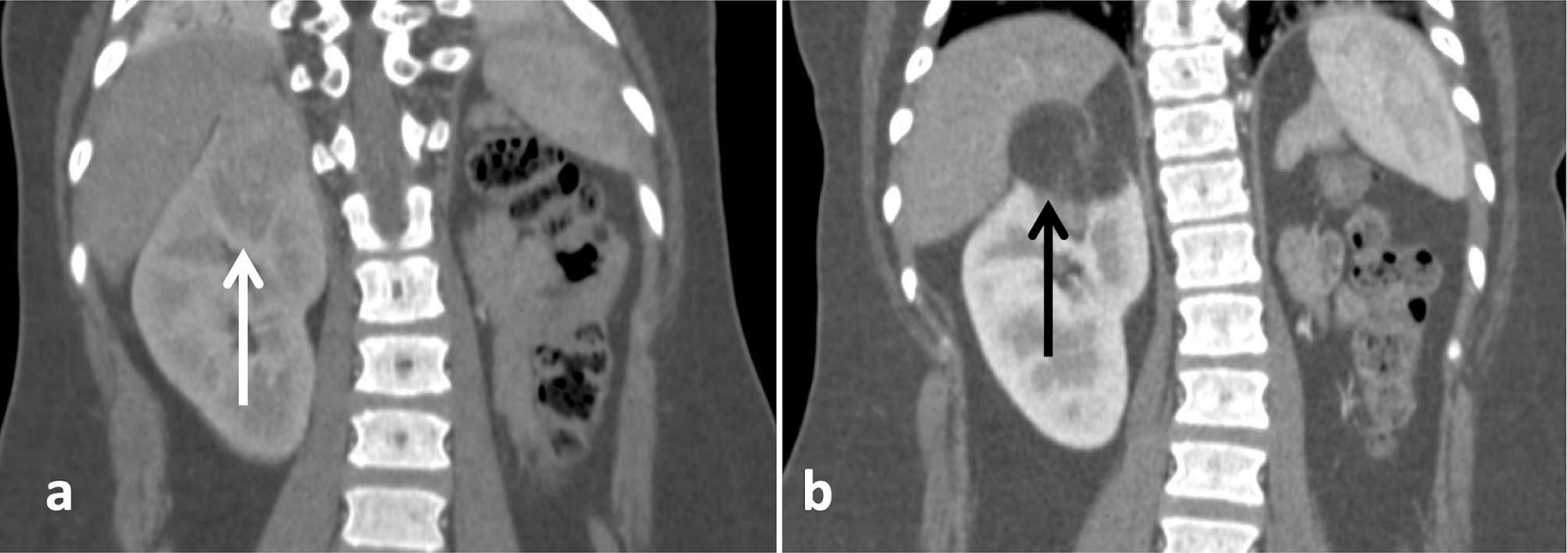
Liposubstitution in angiomyolipoma in response to treatment with sirolimus. Coronal corticomedullary-phase CT images obtained (**a**) before treatment and (**b**) after treatment show the effect of sirolimus treatment on a fat-poor AML in the upper pole of the right kidney (white arrow), revealing the appearance of a fat-rich tumor (black arrow) in substitution to the previous fat-poor pattern.

As a result of these differential effects of sirolimus on the AML compartments, this treatment leads to critical tumor structural changes. Within the scope of visible transformations of the tumor fat profile, some FPTs respond to sirolimus becoming FRTs (Fig. 6).

## Discussion

Renal complications constitute the main cause of death among TSC patients^42–44^. A significant portion of such complications include AML hemorrhage. Although end-stage renal disease is reported at low rates in TSC patients^45,46^, the studied populations are usually young. Notably, up to 40% of the TSC patients develop chronic kidney disease (CKD), exhibiting an estimated glomerular filtration rate equivalent to 30-year-older subjects from the general population^47^. While the pathogenesis of TSC-associated CKD remains not completely understood, several factors are known to contribute to renal function decline, including tumor bleeding, tumor encroaching to normal surrounding renal tissue, renal cystic involvement, focal and segmental glomerulosclerosis and tubulointerstitial disease. A molecular mechanism involving *TSC1* or *TSC2* loss of function in renal parenchyma has also been proposed to play a role in reducing glomerular filtration rate^2,48^. Invasive interventions to prevent tumor hemorrhage, moreover, including partial nephrectomy and selective arterial embolization, can also impact on early loss of renal function as a consequence of loss of functional renal tissue^43^. It should be noted that these invasive procedures are not uncommon in clinical practice^43,47^, given the potential lethality associated with AML hemorrhage. A recent databank study reported that 24.2% of TSC patients had at least one invasive kidney intervention^49^. Because TSC patients usually present multiple AMLs, and the incidence increases with age, such interventions usually cannot treat all lesions. Invasive procedures, therefore, are often repeated along life, leading to increased risk of CKD^43^. Sporadic AMLs, in turn, are usually smaller and single, but may become large and vascularized. Such tumors are also associated with increased risk of bleeding and other complications. Moreover, some patients, such as the cases included in this study, develop large and multiple sporadic AMLs that may or may not be associated with LAM. In such presentations, for reasons similar to TSC, patients are often submitted to invasive therapies that may favor CKD progression.

As a response to this scenario, clinical studies have shown efficacy of mTORi in reducing AML volume, supporting these drugs as first-line therapy for asymptomatic TSC-AMLs larger than 3 cm^48^. The analyzed tumors included TSC-AMLs and sporadic AMLs associated with LAM. Such studies, however, had the primary end-point focused on tumor size reduction, not addressing whether the verified AML shrinkage lowers the bleeding rate and preserves renal function. Interestingly, however, no event of AML bleeding was reported in the extension of the phase 3 study “Everolimus for renal angiomyolipoma in patients with tuberous sclerosis complex or sporadic lymphangioleiomyomatosis (EXIST-2)”^50^, a trial that followed 107 patients treated with everolimus for a median period of 28.9 months. This finding suggests a bleeding protective role of mTORi.

Despite the recognition of this effect of mTORi on AML volume, the response to these drugs has been shown to significantly vary among such tumors^50^. In addition, AMLs are neoplasms histologically complex, with components derived from at least three distinct cellular phenotypes. Based on these two observations, we raised the hypothesis that mTORi might have different effects on distinct tumor components and compartments, and investigated this possibility in AMLs with different constitutions from a series of TSC, LAM and multiple-AML patients. Of note, the elucidation of this effect might not only clarify a complex biological problem but also bring novel, important information to the clinical scenario. Clinical benefits could potentially include the development of predictors for AML response to mTORi as well as risk modifications associated with disease complications.

We created a novel radiologic, CT-anchored AML classification to perform the current analysis, based on previously reported data on tissue-specific ranges of threshold attenuation. As expected, AMLs responded to sirolimus with volume reduction, a response that significantly varied among the tumors. Interestingly, our CT analyses revealed differential effects of sirolimus on AMLs with distinct proportions of fat. Fat-poor AMLs displayed a dramatic volume reduction response to sirolimus while such an effect was much milder in fat-rich tumors. In consistency with these findings, intermediate-fat AMLs presented an intermediate volume decrease behavior between fat-poor and fat-rich tumors. An important clinical derivative of this analysis was the finding that fat poorness predicts a more effective response to sirolimus in AMLs.

Our findings also revealed that the differential effect of sirolimus on AMLs is essentially based on differential effects on specific tumor compartments. Our data showed primary reduction of the fat-poor portions of the tumors. Interestingly, the profound effect of sirolimus on the FPCs and HVCs promoted the transformation of some FPTs into FRTs. The liposubstitution observed in such AMLs represents, in fact, the most striking translation of the remarkable differential actions of mTORi in the different AML compartments. While these effects were previously suggested by an MRI-based study^28^, CT has the advantage of measuring attenuation in standardized units—HU, a property that allows the delimitation of threshold ranges for specific tumor compartments. Moreover, HU attenuation scores confer better reproducibility, enabling the use of such thresholds in different subjects and at any time. In this context, data can be compared irrespective to subject and time. Based on the mentioned threshold ranges, therefore, CT analysis allows volume quantification of each of the tumor components. Of note, the use of contrast media allows the characterization of tumor vascularization, including aneurysms, findings associated with the bleeding risk. In this scenario, our findings unraveled a dramatic decrease of vascular aneurysmatic formations. Since the significant majority of patients with AMLs have an eGFR above 30 mL/min/1.73 m^2^, the use of contrast is not a limitation for them. In line with these conceptual findings, the volume of the AML fat-rich compartment did not decrease, but instead increased, in response to treatment. Our findings of significant reduction of the vascular tumor components in response to sirolimus, in turn, suggest that this treatment is likely protective against AML bleeding.

Notably, although some patients did not present AMLs with all three profiles, the pattern of percent tumor volume response was the same for all but one patient (FPT > IFT > FRT; data not show). Moreover, in spite of significant inter-tumor response variability for the FRC and low variability for the HVC (FRC > IFC > FPC > HVC; data not shown), the average percent volume response of each tumor compartment to sirolimus followed the same pattern in 11 of the 14 patients (HVC > FPC > IFC > FRC, data not shown) and a very minor deviation of such a profile was observed in two cases. While these data suggest decreasing inter-tumor variability from the FRC to the HVC, they also support a conserved inter-patient behavior.

Our data are in line with previous observations that in vitro effects of sirolimus differ among distinct cell lineages^51^ and TSC patients display high levels of the pro-angiogenic molecules VEGF-A and VEGF-D (vascular epithelial growth factors A and D)^52^. mTOR inhibitors, therefore, could differentially act in the different components of AML, with particularly high efficiency on vascular formations. mTOR complex 1 (mTORC1), in fact, is known to drive HIF-1α (hypoxia-induced factor 1α) and VEGF-A signaling via multiple mechanisms involving 4E-BP1 (eukaryotic translation initiation factor 4E-binding protein 1), S6K1 (p70 ribosomal protein S6 kinase 1) and STAT3 (signal transducer and activator of transcription 3), an angiogenic process potentially attenuated by sirolimus^53^. Interestingly, this mechanism is associated with acceleration of endothelial senescence^54^. It is possible, therefore, that the molecular basis of the differential mTORi effects on the AML compartments is based on VEGF downregulation induced by sirolimus through inhibition of mTORC1^55^. Reduction of VEGF-D circulating levels was also reported in patients receiving rapamycin^24^. The remarkable reduction of AML vascular components shown in our study suggests that mTOR activity is fundamental to the maintenance of such structures in AMLs. Moreover, since aneurysms are the main determinant for tumor hemorrhage^29^, the inhibition of maintenance and development of aneurysmatic/ectatic formations induced by mTORi is the likely mechanism responsible for the reduction of bleeding occurrence observed in the EXIST-2 study and its extension^22,50^.

Despite the effects of sirolimus on the AML vascular structures and the strong biological rational, the lack of association between our findings and clinical endpoints, such as tumor hemorrhage rate and eGFR progression, is a limitation of our study. Another potential limitation is the relatively small number of patients/tumors included in the imaging analyses. The consistency of the tumor compartment responses to the treatment with sirolimus, however, strongly supports our conclusions.

Our findings suggest, therefore, that image analysis of AML compartments is likely helpful to predict tumor response to mTORi. Our data revealed, moreover, that treatment with sirolimus not only reduces the tumor size but selectively acts on components associated with more often and more severe clinical complications, such as AML bleeding. Our results provide additional support to the recent recommendation of chronic treatment with mTORi of TSC individuals with AMLs > 3 cm, and suggest that the presence of large aneurysms/vascular formations should be an independent criterium to initiate this therapy. Our newly developed AML evaluation method, moreover, may support the performance of clinical trials associating our findings with meaningful clinical endpoints and the development of an automated in silico method to predict and assess tumor response to mTORi.

## Supplementary Information

Supplementary Information

## Data Availability

The datasets generated and/or analyzed during the current study are not publicly available due to the inclusion of files and images with personal patient information, but are available from the corresponding author on reasonable request.
