# Supplementary material for: The effect of sirolimus on angiomyolipoma is determined by decrease of fat-poor compartments and includes striking reduction of vascular structures
Source: Sci Rep. 2021 Apr 19;11:8493. doi: 10.1038/s41598-021-87930-4 (PMC8055687; doi:10.1038/s41598-021-87930-4)
Supplement: Supplementary file 1 — Supplementary Information [file 41598_2021_87930_MOESM1_ESM.pdf]

# THE EFFECT OF SIROLIMUS ON ANGIOMYOLIPOMA IS DETERMINED BY DECREASE OF FAT-POOR COMPARTMENTS AND INCLUDES STRIKING REDUCTION OF VASCULAR STRUCTURES

Elieser Hitoshi Watanabe, MD, PhD[1]; Fernando Morbeck Almeida Coelho, MD[2]; Hilton Leão Filho, MD[2]; Bruno Eduardo Pedroso Balbo, MD, PhD[1]; Precil Diego de Miranda de Menezes Neves, MD[1]; Fernanda Maria Franzin, PhD[1]; Fernando Ide Yamauchi, MD[2]; Luiz Fernando Onuchic, MD, PhD[1]*.

[1]*Division of Nephrology, Department of Medicine;*

[2]*Division of Radiology, Department of Radiology and Oncology;*

*University of São Paulo School of Medicine, São Paulo, Brazil.*

## Supplementary Figure Legend

**Figure S1:** Image-based assessment of sirolimus effects on AMLs. (a-h) Histogram analysis of the AML displayed in Figure 2 shows pixel densities below -30 HU (fat-rich compartments) in a (pre-treatment) and e (post-treatment), ≥-30 and <30 HU (intermediate-fat compartments) in b (pre-treatment) and f (post-treatment), ≥30 and <100 HU (fat-poor compartments) in c (pre-treatment) and g (post-treatment), and ≥100 HU (highly-vascularized compartments) in d (pre-treatment) and h (post-treatment).

**Figure S2.** Percent total volume response to sirolimus between TSC-associated and sporadic AMLs. AML: angiomyolipoma; TSC: tuberous sclerosis complex. Comparison performed with the Mann Withney U test.

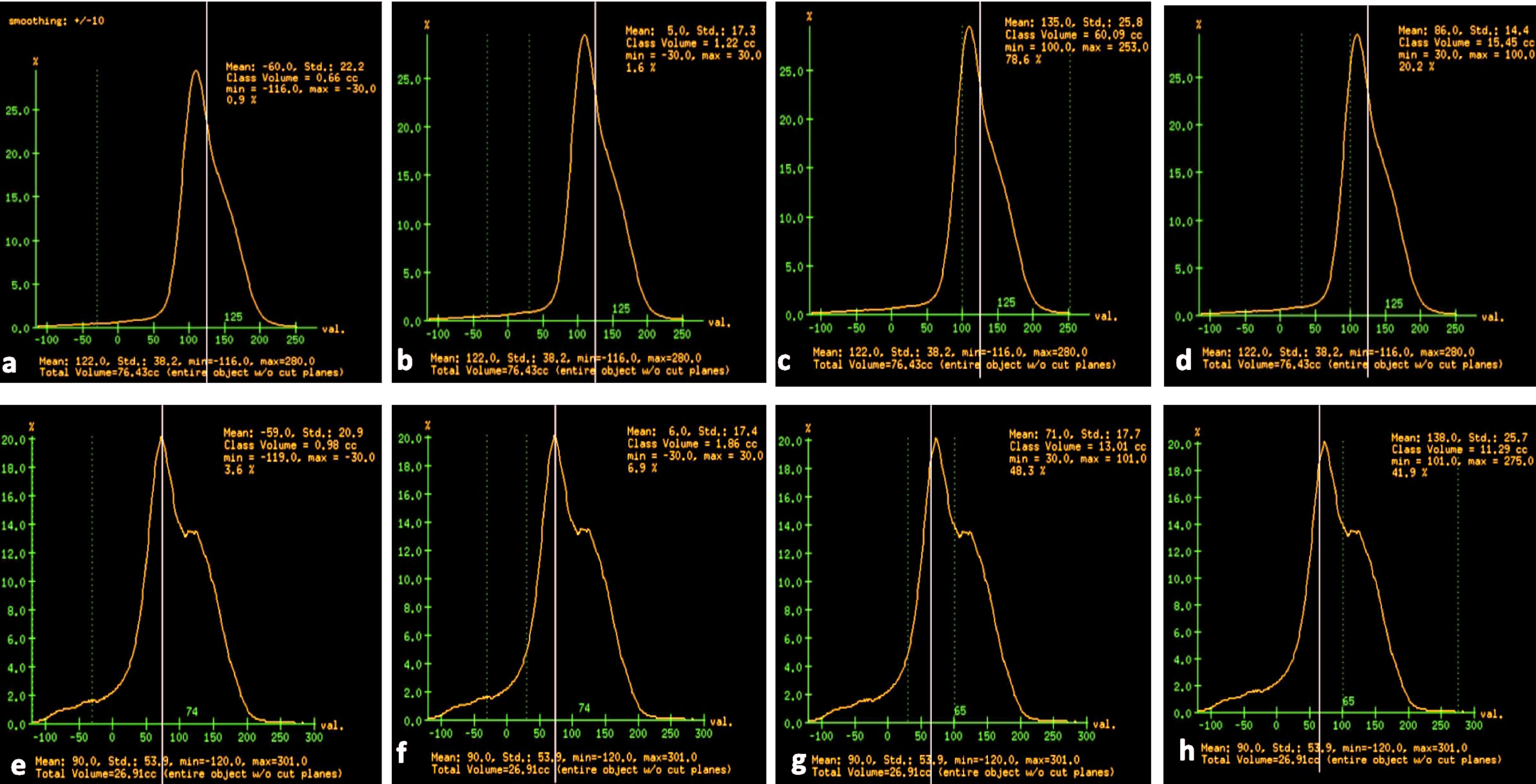
a
smoothing: +/-10
Mean: -60.0, Std.: 22.2
Class Volume = 0.66 cc
min = -116.0, max = -30.0
0.9 %
125
Mean: 122.0, Std.: 38.2, min=-116.0, max=280.0
Total Volume=76.43cc (entire object w/o cut planes)
b
Mean: 5.0, Std.: 17.3
Class Volume = 1.22 cc
min = -30.0, max = 30.0
1.6 %
125
Mean: 122.0, Std.: 38.2, min=-116.0, max=280.0
Total Volume=76.43cc (entire object w/o cut planes)
c
Mean: 135.0, Std.: 25.8
Class Volume = 60.09 cc
min = 100.0, max = 253.0
78.6 %
125
Mean: 122.0, Std.: 38.2, min=-116.0, max=280.0
Total Volume=76.43cc (entire object w/o cut planes)
d
Mean: 86.0, Std.: 14.4
Class Volume = 15.45 cc
min = 30.0, max = 100.0
20.2 %
125
Mean: 122.0, Std.: 38.2, min=-116.0, max=280.0
Total Volume=76.43cc (entire object w/o cut planes)
e
Mean: -59.0, Std.: 20.9
Class Volume = 0.98 cc
min = -119.0, max = -30.0
3.6 %
74
Mean: 90.0, Std.: 53.9, min=-120.0, max=301.0
Total Volume=26.91cc (entire object w/o cut planes)
f
Mean: 6.0, Std.: 17.4
Class Volume = 1.86 cc
min = -30.0, max = 30.0
6.9 %
74
Mean: 90.0, Std.: 53.9, min=-120.0, max=301.0
Total Volume=26.91cc (entire object w/o cut planes)
g
Mean: 71.0, Std.: 17.7
Class Volume = 13.01 cc
min = 30.0, max = 101.0
48.3 %
65
Mean: 90.0, Std.: 53.9, min=-120.0, max=301.0
Total Volume=26.91cc (entire object w/o cut planes)
h
Mean: 138.0, Std.: 25.7
Class Volume = 11.29 cc
min = 101.0, max = 275.0
41.9 %
65
Mean: 90.0, Std.: 53.9, min=-120.0, max=301.0
Total Volume=26.91cc (entire object w/o cut planes)
%
val.

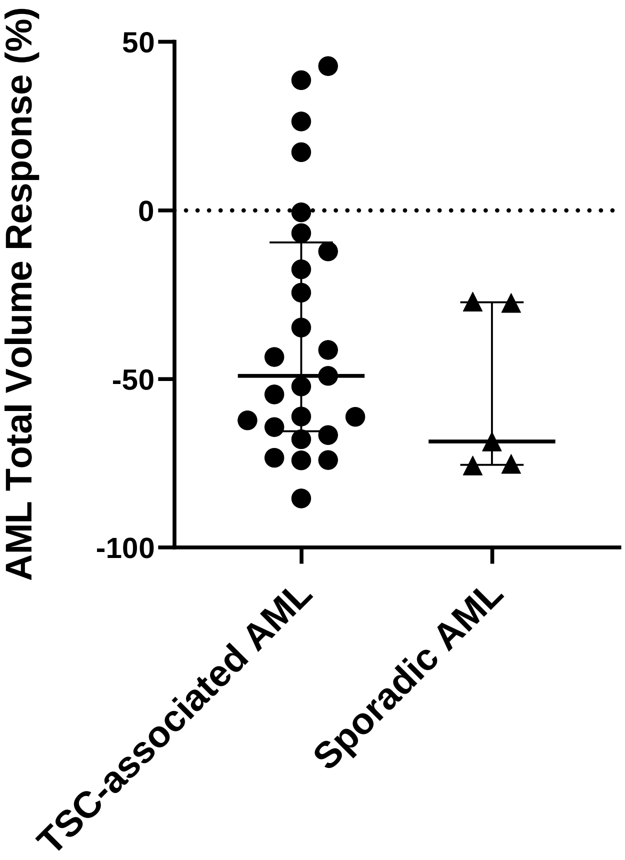
AML Total Volume Response (%)
50
0
-50
-100
TSC-associated AML
Sporadic AML
